# First person – Claudia Figueroa-Romero and Benjamin Murdock

**DOI:** 10.1242/dmm.042952

**Published:** 2019-11-15

## Abstract

First Person is a series of interviews with the first authors of a selection of papers published in Disease Models & Mechanisms (DMM), helping early-career researchers promote themselves alongside their papers. Claudia Figueroa-Romero and Benjamin Murdock are co-first authors on ‘ Temporal evolution of the microbiome, immune system and epigenome with disease progression in ALS mice’, published in DMM. Claudia is an Assistant Research Scientist in the lab of Eva L. Feldman at the Department of Neurology, University of Michigan, USA, investigating how life-long environmental exposures, including the microbiome, may play a role in the development of late-onset neurodegenerative diseases, such as ALS. Benjamin is a Research Assistant Professor in the lab of Eva L. Feldman, examining the role of the immune system in ALS progression.


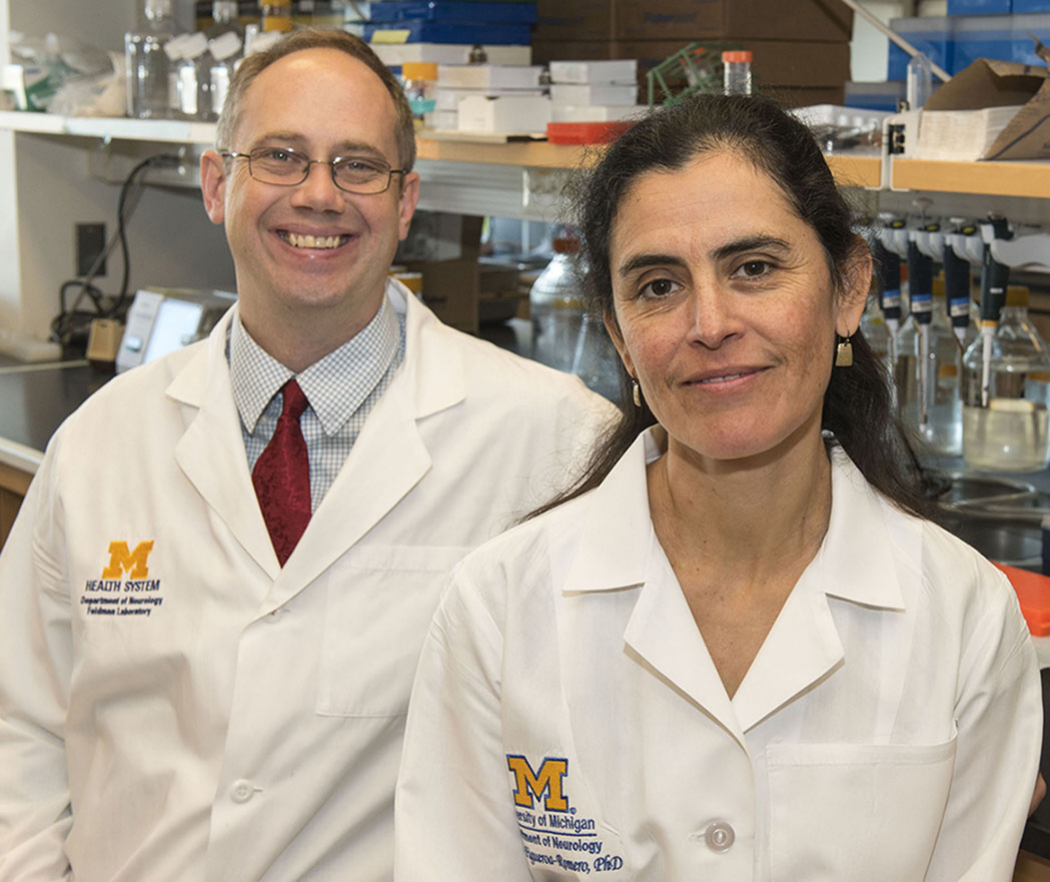


**Benjamin Murdock and Claudia Figueroa-Romero**

**How would you explain the main findings of your paper to non-scientific family and friends?**

BM+CFR: We find that changes in immune cell types, gut bacteria and epigenetic marks are associated with the progression of ALS in mice. However, these changes do not occur all at once. By examining the timing of these changes, we can begin to understand how different systems impact one another during ALS. For instance, many of the changes in bacterial populations precede changes in the immune system, suggesting that the microbiome may have downstream effects on immune cell composition during disease.

“The microbiome may have downstream effects on immune cell composition during disease.”

**What are the potential implications of these results for your field of research?**

BM+CFR: Both bacterial communities and immune changes have been previously implicated in ALS progression. Defining the temporal occurrence and interrelation of these pathological processes may identify specific windows when the disease may be treated with specific therapies. In addition, because of the strong link between the microbiome and the immune system, we may be able to modulate the impact of the immune system in ALS by changing the microbiome: probiotics, fecal transplants, etc. may be viable therapeutic options to slow ALS progression.

**What are the main advantages and drawbacks of the model system you have used as it relates to the disease you are investigating?**

BM+CFR: The SOD1^G93A^ mouse model is a robust and highly reproducible ALS animal model, resulting in progressive motor neuron degeneration, increasing muscle weakness and eventual paralysis. As the SOD1 mouse is a knock-in mouse using a mutated copy of the human SOD1 gene, researchers have the option of using ‘low copy number’ mice, as we did in our study. Fewer copies of the mutated gene slow disease progression, resulting in a greater number of time points that allowed us to study changes over a longer period of time.

The SOD1 model does have its drawbacks, however. Foremost among them is the fact that it models a relatively small percentage of ALS patients: familial ALS cases represent only 10% of ALS cases arising from genetic mutations, the rest develop sporadically in ways we do not yet understand. One of the main challenges in ALS research is the lack of mouse models of sporadic ALS.

**What has surprised you the most while conducting your research?**

BM+CFR: We find it fascinating how well-orchestrated the cellular mechanism changes are during disease progression and how early in life those changes occur. We were also surprised by the number of different bacterial groups that showed significant correlation with immune and phenotypic changes. I think we expected several bacterial groups to correlate with other changes, but not several dozen. On one hand, it's exciting to find that the microbiome seems to be playing a role in disease, but at the same time the sheer complexity of the changes is somewhat intimidating.

“We find it fascinating how well-orchestrated the cellular mechanism changes are during disease progression.”

**Describe what you think is the most significant challenge impacting your research at this time and how will this be addressed over the next 10 years?**

BM+CFR: We currently don't know what causes ALS in most patients. It is thought that ALS develops in a manner similar to cancer, with multiple ‘hits’ throughout life leading to disease. However, that lack of a single, driving disease factor, along with the highly variable nature of disease presentation, makes ALS research challenging. How representative are our mouse models of human disease? How similar are patients with limb or bulbar onset? How similar are fast- or slow-progressors? Are there gender differences in disease mechanisms? If our results in animal models are translatable to humans, how do we identify early-life pathological processes in humans that will lead to late-onset neurodegeneration? All of these questions muddy the waters; however, we and many other groups are looking to examine these issues in the years to come.

**What changes do you think could improve the professional lives of early-career scientists?**

BM+CFR: I think formalized training would help in a lot of areas of science, particularly for young investigators. Although scientists are trained how to think and how to do research, other important career aspects are learned in an ad hoc manner or are even improvised. Leadership training, communication, teaching skills, networking skills, finances, etc. are learned via trial and error. A standardized curriculum which includes these areas would be hugely beneficial.

“Formalized training would help in a lot of areas of science, particularly for young investigators.”

**What's next for you?**

BM+CFR: The findings from this study implicate that dysregulated microbiome and immune homeostasis occurs in early life in familial ALS. We would like to identify the molecular players facilitating the cross-talk between the host and the gut microbiota to understand what specific mechanisms contribute to disease onset and progression in order to develop specific therapeutic targets for ALS. Our ultimate goal is to repurpose existing pharmaceuticals and treatment methods to slow disease progression in ALS patients. To this end, we have several ongoing ALS studies that use clinical human samples, mouse models and *in vitro* assays to identify immune cells and bacterial populations involved in disease progression. We are currently testing a candidate drug for use in ALS: our *in vitro* studies show that it reduces the activation and activity of cell types associated with disease. We will soon be examining its effects in pre-clinical mouse models of ALS.
